# Correction: Gut carriage of antimicrobial resistance genes in women exposed to small-scale poultry farms in rural Uganda: A feasibility study

**DOI:** 10.1371/journal.pone.0252261

**Published:** 2021-05-20

**Authors:** Ana A. Weil, Meti D. Debela, Daniel M. Muyanja, Bernard Kakuhikire, Charles Baguma, David R. Bangsberg, Alexander C. Tsai, Peggy S. Lai

In the Funding section, the URL for the funder Friends of a Healthy Uganda is incorrect. The correct Funding statement is as follows: Funding was provided by National Institutes of Health grants K23 ES023700 (PSL), K23 MH096620 (ACT), and K08AI123494 (AAW) (https://www.nih.gov/), Harvard School of Public Health-National Institute of Environmental Health Sciences and Center for Environmental Health (P30ES000002) Pilot Project Grant (PSL) (https://www.hsph.harvard.edu/niehs/), American Lung Association Biomedical Research Grant RG-346990 (PSL) (https://www.lung.org/), Harvard Catalyst (UL1 TR001102) Early Clinical Data Support Pilot Grant (PSL) (https://catalyst.harvard.edu/), and Friends of a Healthy Uganda (DRB, ACT). The funders had no role in study design, data collection, analysis, decision to submit the work for publication, or preparation of the manuscript.
