# Supplementary figures and images for: Gut carriage of antimicrobial resistance genes in women exposed to small-scale poultry farms in rural Uganda: A feasibility study
Source: PLoS One. 2020 Jun 11;15(6):e0229699. doi: 10.1371/journal.pone.0229699 (PMC7289395; doi:10.1371/journal.pone.0229699)

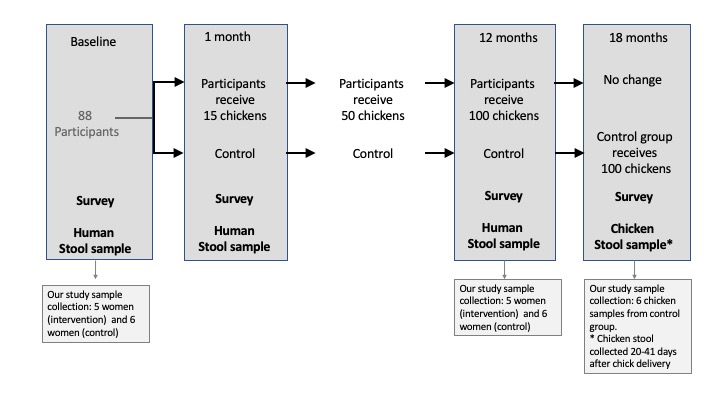

Supplement: S1 Fig — (JPG) [file pone.0229699.s001.jpg]
