# Supplementary material for: Gut carriage of antimicrobial resistance genes in women exposed to small-scale poultry farms in rural Uganda: A feasibility study
Source: PLoS One. 2020 Jun 11;15(6):e0229699. doi: 10.1371/journal.pone.0229699 (PMC7289395; doi:10.1371/journal.pone.0229699)
Supplement: S1 Method — (DOCX) [file pone.0229699.s003.docx]

**SUPPLEMENTARY METHODS**

Survey questions were administered in Runyankole (the local language) by trained research assistants on password-protected tablets. Specific questions used in this study are listed below.

**PARTICIPANT MEDICATION USE**

1. Have you taken any medicines in the past 3 months? *By medicine, I mean medicine prescribed by a doctor or medicine you buy in a pharmacy. Do not count traditional medicines.*

a. Were any of the medicines a medicine to treat infection (an antibiotics)?

b. Were any of the medicines a steroid medicine? Some examples of steroids are prednisone, methylprednisolone, solumedrol.

c. Were any of the medicines a medicine you breath in (inhaler or nebulizer) to treat a lung problem?

2. Do you know the names of the medicine you are taking?

a. If yes, please list the names of the medicine you are taking. *Please list one medicine per line.*

**CHICKEN CARE**

1. How many chickens are currently in the chicken coop?

2. How many of the chickens in the coop currently are broilers?

3. How many weeks old are the chickens currently?

4. Does the participant also own village chickens? *By village chickens, I mean chickens that do not need the special care that broilers and layers need, like a chicken coop, feed you have to buy from a supplier, and medicines.*

5. Are your chickens currently brooding?

6. Have your chickens received vaccines against Newcastle?

7. Have your chickens received vaccines against Gumboro?

8. Have you ever given your chickens any medicines?

9. Which of the following medicines have you given your chickens? Please select all given.

a. Coccid

b. Alfacycline

c. Oxiveto

d. Alamycin chick formula

e. Alamycin egg formula

f. Other: Please specify.
