# Supplementary material for: Gut carriage of antimicrobial resistance genes in women exposed to small-scale poultry farms in rural Uganda: A feasibility study
Source: PLoS One. 2020 Jun 11;15(6):e0229699. doi: 10.1371/journal.pone.0229699 (PMC7289395; doi:10.1371/journal.pone.0229699)
Supplement: Attachment — Submitted filename: R2R.docx [file pone.0229699.s004.docx]

April 23, 2020

Dear Dr. Karunasagar

We thank the reviewers for the thoughtful comments on our manuscript, “Gut carriage of antimicrobial resistance genes in women exposed to small-scale poultry farms in rural Uganda: a feasibility study”. We feel the comments have substantially improved the manuscript. Major changes made are:

1. Changes to the text highlighting the limitations of using two stool samples from the human cohort to make definitive statements about acquisition of AMR genes
2. Clarification of the timing of chicken stool collection. Although chicken stool was collected 18 months after randomization, it was collected between 20 – 41 days after the control participants received these chickens. This is because chickens were not distributed to control participants until approximately 17 months after randomization.
3. Addition of a permutational analysis of variance (PERMANOVA) analysis comparing similarities/differences in AMR patterns between control and intervention groups at baseline and one year after randomization
4. Revision of the human-chicken centroid analysis to account for repeated measures. Instead of using linear regression we have used a mixed effects model to account for repeated measures in an individual.

Below we have provided a point-by-point response to individual reviewer comments.

--

**Reviewer #1: Weil et al., provide a manuscript detailing the feasibility of conducting a delayed intervention trial with the objective of measuring the patterns of fecal AMR genes in chicken farmers before and after poultry introduction to a “farm” or household. To my knowledge, the application of a delayed intervention RCT design for a longitudinal study of antimicrobial resistance maker gene prevalence in humans and livestock is novel. This feasibility study identifies many factors that should be considered in design and implementation of such a study. Many of the critical factors identified are omitted in this study, and some additional factors in data analysis are not mentioned. To my knowledge, using a Jaccard dissimilarity index is a novel approach to estimating shared antimicrobial resistance markers in populations or groups.**

**Major concerns:

1. The objective or hypothesis of the study is unclear. Please revise the sentence at the end of the introduction section to provide a more comprehensive description of study goals or objective(s) or any specific hypothesis.**

**Response:** We have revised the manuscript introduction to clarify the objective of the study.

**2. The unbalanced design by not sampling the chickens at the time of introduction in both the intervention and control group, and not sampling humans at the same time as chickens is a major limitation and should motivate a more conservative interpretation of the results. While you recognize this limitation in the discussion, in my opinion it seems biologically unjustified to test associations between the resistance genes found in the human groups and the chickens provided to the control group 6 months after the human samples were collected. As you note in the introduction temporal aspects and history of exposure are critical. Further, the study design seems to assume a single chicken sample, taken 6 months after the human one year samples, is a realistic representation of the chicken samples provided to the intervention group. Previous animal (and human) studies have shown resistance markers change over time and especially in relation to antibiotic exposure (e.g. tetracycline exposure in the brooders) and life cycle or management changes.**

**Response:** We appreciate the reviewer comments regarding study design rationale and limitations. We have added clarifications on several of these points to the manuscript. Some study design concerns, such as sampling of the humans and chickens at the same time, are not addressable at this time, and we have therefore added to the study design limitations referenced in the discussion. Please note that although the chicken stool samples were collected 18 months *after randomization*, that does not mean chickens were introduced at 12 months *after randomization* in the control group. At the time of chicken stool collection, chickens had only been in contact with their human caretakers for between 20 and 41 days.

**3. In my opinion, there are too many places in the manuscript where you imply possible chicken to human transmission, including in the approach to analysis, the presentation of results, the interpretation/discussion, and the conclusions. Is this an objective of the study? (e.g. lines 48, 172, 254) Please refine the description of the study objectives and in doing so justify the approach. Specifically comment on why you did not collect base-line and one year samples from the poultry prior to and after introduction to the households in both groups, and why you did not collect 18 month samples in humans. Please refine the description in the methods describing the duration of contact between humans and poultry at the 18 month time point prior to chicken sample collection.**

**Response:** We have revised the manuscript per the reviewer comments and removed any implication of chicken to human transmission, and this issue is also discussed directly in our limitations section. We have also refined the study objectives, and explained the rationale for the lack of baseline samples from chickens or 18 month samples in humans in the methods section.

**4. An important aspect of the feasibility analysis would be an analysis of the variability in gene marker occurrence in the control and intervention groups over time and between subjects within groups. Please explore this variability using the data available and describe how such variability would influence future study design (e.g. power calculations to explore effect size estimates). While you have commented on the small sample size limitation in the discussion, you did not specifically comment on the infrequent repeated measures and evaluating variability in outcomes over time and power needed to discriminate between noise (random variation) and true trends over time. In my opinion, 2 time points are insufficient to make inferences regarding trends over time. Longer term surveys of antimicrobial resistance trends demonstrate sample frequency influences interpretation.**

**Response:** We have added to the limitations that our analysis is based on only 2 human timepoints and accompanying lack of power associated with this limitation. The variability in AMR gene patterns and a statistical analysis are further addressed in point 6 below. The raw data is provided as a supplemental file in case a reader wants to attempt power calculations for a future study; we would caution against applying power calculations from our study results to a completely different setting as there may be context-specific differences in AMR gene patterns.

**5. Because it appears you did not account for within subject repeated measures, nor did you appear to account for clustering (the hierarchical nature of subjects and chickens within households and within villages), in the discussion, please comment on: 1) longitudinal studies that include repeated measures within subjects should include assessments of correlation within subjects and models assessing associations should account for repeated measures and autocorrelation, 2) the potential effect of clustering within households and villages (e.g. humans in the same villages (or connected households within villages) may have greater resistance gene similarity compared to humans from other villages, independent of the chicken exposure treatment). For example, inclusion criteria for this study was the household must have at least 1 child under the age of 5. What if the household also had school-aged children who attend school? This is just one obvious example (of many) that creates a possible contact network between households (not to mention within village shared water sources, which you mentioned, or shared latrines or shared foods or shared food production environments).**

**Response:** We thank the reviewer for the opportunity to clarify. We recruited only 1 participant per household. Within Nyakabare parish, there are 7 villages. Each intervention participant was from a distinct village. Control participants were chosen to be from the same village as a matched intervention participant. That does not preclude the possibility of social contact but there is some geographic distance between villages.

With the small number of participants, a model that accounts for clustering of subject within village will not run because there is not enough data to create correlation structures, however, we can adjust for repeated measures in an individual. Therefore we have revised the analysis determining the distance between human and intervention control group AMR patterns to the chicken centroid using mixed effects models rather than linear regression as the reviewer suggests.

These limitations to the study design are emphasized in our discussion.

**6. Building on the previous comment, how do you control for the possibility of community acquired resistance genes in this study? Please comment on the limitations in this study design? Does this preliminary work suggest it would be possible to replicate the study across multiple villages? A challenge with longitudinal infectious disease studies is when intervention and control groups co-mingle and you cannot control for the possible contact and transmission between the intervention and control groups. This issue should be addressed in additional analyses of the data and in the discussion; for example the human baseline resistance gene profiles in the control group (or possibly a pooled baseline of the time zero control and intervention groups) could be used as the centroid to test the similarity between intervention or control subjects at one year or chickens at 18 months compared to controls (or pooled data) at baseline. I would recommend conducting a similarity analysis to first demonstrate that the baselines in the 2 human groups are not different (not sure how one might do tis in the absence of replication), before pooling the baseline data for subsequent analysis. Given that 10 of the 32 markers were found in only chickens and 13 of the 32 markers were found in both chickens and humans, and the majority of the markers 22 were found in humans, I suspect there may be greater similarity in markers found in the one year samples compare to base-line humans than in markers found in chickens compared to base-line humans. And there may be an issue with the uneven frequency of sampling (i.e. lower overall sample numbers in the chickens results in a under-estimate of the true frequency of resistance markers in this population.)**

**Response:** To address the question of whether AMR gene profiles were different between intervention and control groups at baseline and 12 months after randomization, used permutational analysis of variance (PERMANOVA) on the Jaccard distance to determine whether the centroids of the intervention and control groups differ at baseline and 12 months after randomization in the control vs. intervention groups. At baseline, there was no statistically significant difference in AMR resistance patterns between control vs intervention groups (PERMANOVA R2 = 0.081, p = 0.61) whereas at 12 months there is a trend towards a difference in AMR patterns between these two groups (PERMANOVA R2 = 0.186, p = 0.09). Our study was not powered to detect differences in AMR patterns but these findings do suggest the possibility that in a larger study, we may have been able to demonstrate that there were no differences in AMR resistance between these groups at baseline, but at 12 months after chicken exposure such differences may have been identified, with the caveats of sampling the reviewer highlights.

We cannot control for the possibility of community acquired resistance genes in our study. This is highlighted in the discussion portion of our manuscript. This aspect would be critical for future rigorous studies to identify sources of AMR genes in humans, livestock, and the environment.

**7. Methods – please add more details on the methods of recruitment of the women in this study as a subset of the original RCT. Was this a convenience sample subset of the original eligible subjects? Please add details as to whether stool sample collection was included in the parent RTC plan, or if the sample collection and outcome measures in this feasibility study were a post-hoc additional to the original study.**

**Response:** We appreciate the importance of adding additional detail to the recruitment information in this study. We have added the following to the methods; “We recruited participants from an existing randomized clinical trial (RCT) of small-scale chicken farming (ClinicalTrials.gov Identifier: NCT02619227) [15]. Participants were a convenience sample chosen from the original trial and assessment of antimicrobial resistance gene carriage was added as a post-hoc aim.”

The original RCT was designed as an economic intervention focused on food insecurity, which is unfortunately widely prevalent in the study population. We saw the opportunity to perform a feasibility study based on the existing RCT infrastructure, and to share our experience so that a future more definitive trial focused on the important topic of antimicrobial resistance gene transmission between humans, animals, and their environment can be designed and implemented.

**8. Please add some discussion (at least) and possibly some analysis of the Jaccard dissimilarity index approach to estimating shared antimicrobial resistance markers in populations or groups. Are there other studies that have applied this approach? Are there any studies that have compared using this summary index in a regression model with a more traditional approach using a binary outcome such as presence or absence of a gene marker or class of genes with corrections for multiple comparisons?**

**Response:** With high-dimensional antimicrobial resistance data, a number of studies have used dissimilarity measures such as the Bray-Curtis, Jaccard, or other beta diversity metrics used in microbial ecology. This is to address the large p small n problem, where the number of features (p here represents genes) overwhelms your sample size (n, here number of subjects in the study). To use more traditional approaches like logistic regression with a strict Bonferroni correction of the p-values will require sample sizes that are simply not feasible in terms of manpower, resources, and budget. In high dimensional statistics, the use of simulation-based methods such as permutational analysis of variance (PERMANOVA) on some beta diversity or distance metric is an acceptable statistical approach. For a more thorough review of this approach please see the excellent “Modern Statistics for Modern Biology” book by Susan Holmes and Wolfgang Huber.

Example citations using this approach below, also now cited in the manuscript.

Gasparrini et al. Persistent metagenomic signatures of early-life hospitalization and antibiotic treatment in the infant gut microbiota and resistome. Nat Microbiol. 2019 Dec;4(12):2285-2297. PMID: 31501537. PMCID: PMC6879825

Agga et al. Antimicrobial-Resistant Bacterial Populations and Antimicrobial Resistance Genes Obtained from Environments Impacted by Livestock and Municipal Waste. PLoS One. 2015 Jul 21;10(7):e0132586. PMID: 26197056. PMCID: PMC4510610.

**9. Are chickens ever free-ranging during the course of the study? How long was it until slaughter for the chickens provided to the intervention group? Were slaughtered chickens sold in the village? How frequently were intervention household chickens purchased by (or shared with) control households? My experience with poultry introduction programs in rural communities which provided coup construction in addition to animals, is that the poultry are often raised extensively, (i.e. allowed to forage outside the coup areas) for significant periods of time during the day or their lifetime, and may be returned to owner’s coups at night. Chickens will then forage in cattle (and other livestock) dung and the broad environment (including human waste and especially any human food waste such as vegetable scraps which may have fecal/manure contaminants and be a source of resistance genes). Thus the chicken diet and potential exposure is not well controlled in many rural systems, and chickens may simply be a sentinel for common environmental exposure risk. Were these variables considered and controlled for in this study? If appropriate this issues should be addressed in the discussion section.**

**Response:** A requirement of study participation was construction of a chicken coop to confine the chickens; this information was added to the methods section. The primary study was designed as a series of microloans. Per study protocol, the time to slaughter was 6 – 8 weeks though participants were given two extra weeks to sell off the birds and pay back the loan. These details have been further highlighted in the methods section.

**10. In my opinion, the authors over emphasize speculative transmission (based on similarity) of resistance markers between humans and chickens, when alternative transmission dynamics, independent of poultry contact, such as human to human or human to environment to human, can explain the observed data and are not explored in the analysis – if the intervention subjects and the control subjects are considered to belong to the same population (i.e. from the same village) then at base-line almost all of the observed genes present in the human population are also found in either the intervention or control group at 12 months. From table 2 and table S1, it appears 4 (12%) of 32 (did I count correctly?) total genes were found in either the intervention or control groups at 1 year [aaC2, AAC(6)-Ib-cr, CFE-1 and LAT] but not at baseline, and none of these markers were found in chickens. It appears that the majority of genes found in chickens at 18 months were pre-existing in humans at baseline (i.e. before introduction of chickens for this trial), except for 10 genes (31% of all genes), [vanB, vanC, msrA, erm A, ermC, SHV(238S240E), SHV(238S240K), OXA-10 group, and OXA-58 group, QnrB-8 group], which were only found in chickens. There are many examples where a marker was found in one or both groups at one year but was not observed in chickens [AAC(6)-Ib-cr, aacC2, CTX-M-9 Group, SHV(156D), ACC-3, ACT 5/7 group, CFE-1, LAT, QnrB-1 group ] including at least 4 elements found in the control group prior to direct chicken contact. Further there are many examples were a resistance marker existed in baseline samples in one group and emerged in the other group at 12 months [CTX-M-1 Group existed in intervention at base then found in control at 1 year; SHV, SHV(238G240E), ACT 5/7 group, QnrB-5 group, QnrS in controls at base and found in intervention at 1 year]. Overall these findings suggest to me there is more similarity between human samples in the 2 groups at baseline and one year, than there is between human and poultry samples. While you discussed this concepts, with some specific examples in the discussion section. These associations should be explored more thoroughly.**

**Response:** We appreciate the reviewer’s comments here to help us to improve the manuscript. The reviewer is correct that over time, there was emergence of specific AMR genes in both control and intervention group, which we feel represents the background AMR fluctuations in the community that are unrelated to our study. As noted in point 6 above, using permutational analysis of variance (PERMANOVA) on the Jaccard distance between baseline samples from both control and intervention groups, we cannot detect a statistically significant difference in AMR resistance patterns between these groups (PERMANOVA R2 = 0.081, p = 0.61) whereas at 12 months there is a trend towards a difference in AMR patterns between these two groups (PERMANOVA R2 = 0.186, p = 0.09).

In the manuscript we highlight the limitations of only two sampling time points in humans, highlight the possibility of community reservoirs of transmission, and de-emphasize the possible transmission between

**Line 34 – eliminate “growth promoters” – all antimicrobial use in livestock production likely contributes to emergence and selection of AMR genes in livestock and possibly subsequently to humans either through direct exposure to animals and their housing environments or through indirect exposure through the food chain (i.e. contaminated food products harvested from animals).**

**Response:** This was done as suggested.

**Line 34 and line 77 – delete “major” – this is subjective; I think many believe, and some research data would suggest, the major contributor to acquisition of AMR genes in humans is antimicrobial use in humans. See for example the systematic review and meta-analysis by Tang et al. (Lancet Planet Health 2017;**
[**http://dx.doi.org/10.1016/S2542-5196(17)30141-9**](http://dx.doi.org/10.1016/S2542-5196(17)30141-9)**).**

**Response:** This was done as suggested.

**Line 37 – delete “from”**

**Response:** This was done as suggested.

**Line 47 – revise order, this is confusing as it does not represent what I interpret is the actual study design and sampling protocol… it might read “After one year of exposure to chickens, seven new AMR genes were detected in the intervention group at one year, while six new AMR genes were detected in the control group at one year and 6 months prior to when this group received chickens.”**

**Response:** This sentence was deleted.

**Line 49 – revise “Women who had direct contact with the chickens sampled in the study had greater similarities in AMR resistance gene patterns to chickens than those who did not have direct contact with chickens sampled.” This sentence is misleading. In your model using the similarity outcome, at base-line (i.e. before any contact) and at one year (after contact), women with direct contact with chickens during the year (i.e. the intervention group) actually had lower similarity to chicken samples (taken at 18 months) than the control group (who had no direct household chicken contact at either baseline or one year).**

**Response:** We apologize for the confusions. The chickens sampled in this study were from the control group, who received their chickens at the end of the study. Therefore the control women are the ones that had direct contact with the chickens sampled in the study. The abstract has been revised as follows: “Women in the control group who had direct contact with the chickens sampled in the study had greater similarities in AMR patterns to chickens than those in the intervention group who did not have direct contact with chickens sampled (p = 0.01) although at one year there was a trend towards increased similarity in AMR patterns between chickens and humans in both groups at one year (p = 0.06). “

**Line 54 – revise - there were not chicken samples at both baseline and follow up time-points. The chicken samples are temporally distant from the human samples and it is not clear the length of contact time between humans and chickens before the chicken samples were taken at 18 months .**
**Response:** We have removed this line.

**Line 68 – “The problem is accentuated…” The information in this sentence seems logical, but without data, is speculative – please revise this sentence. Please clarify, is this a hypothesis statement for future studies or can you provide a reference supporting this statement with empirical data?**

**Response:** We have revised the sentence as follows. “This problem may be accentuated in resource-limited settings due to a likely higher burden of infectious disease, little to no antimicrobial stewardship, less resources for microbiology testing, and possible limited access to antibiotics targeting highly resistant pathogens.”

The issue with performing AMR studies in resource-limited settings is that most clinical microbiology labs in these locations do not have the consumables and equipment needed for rigorous AMR determination.

**Line 93 – please end the introduction with a sentence describing the actual objective(s) (or hypothesis?) of the study – was the objective simply to determine the feasibility of conducting a longitudinal study of AMR gene patterns in chicken farmers and their poultry in a delayed intervention RCT? Or was the objective to test a specific hypothesis? From the current sentence, it seems the objective was to determine patterns of AMR genes in stool of chicken farmers before and after poultry introduction, but the methods appear to go beyond this descriptive objective to test associations between resistant elements found in poultry and their caregivers. Yet, as discussed under general concerns, the study design for this test of association is incomplete and the statistical approach ignores the hierarchical nature (clustering) and repeated measures (correlation) in the sample populations.**

**Response:** We have added the following line. “In this study we determined the feasibility of a longitudinal study of AMR gene patterns in a subset of participants enrolled in a delayed intervention RCT of small-scale chicken farms in rural Uganda.”

**Line 107 - What was the duration of the brooding period, and more specifically what was the duration of feeding tetracycline-containing dietary supplements? Were these supplements provided in water or in solid feed? What was the target dose of tetracycline as fed per kilogram animal unit?**

**Response:** We have added the following details to the methods:

“Participants also routinely administered dietary supplements to chicks in their drinking water during the brooding period as part of a protocol to boost growth. This included two oxytetracycline-containing medication; Alamycin chick formula given for the first 2-3 weeks of brooding and Oxiveto given weekly for four weeks. In addition, Coccid (which contains amprolium) was given once weekly for four weeks to prevent coccidiosis.

**Line 110 – how are the survey data being used in this study? In particular the records of antimicrobial use for the women study participants and for treatment of all livestock on the farms. Was this a new survey developed only for this feasibility study, or were data extracted from a larger survey developed for the original larger RTC. Please provide a copy of the survey tool in the supplemental data, or provide a link to a permanent archive for the survey tool.**

**Response:** The survey data was only used in this study to detect self-reported antibiotic use among the study population, and this data was extracted from the larger RTC survey. We have added these details to the methods and we have added the survey tool to the supplemental information.

**Line 113 – modify figure S1 to indicate the actual number of participants sampled in the 2 arms of the this feasibility study**

**Response:** This was done as suggested.

**Line 115 – so at 18 months, when the control group receives their first batch of brooding chicks (which were receiving medicated feed or feed supplements at the time of sampling?) you take chicken fecal samples. This seems to be an apples and oranges comparison group, because you have longitudinal samples from the humans and a single point in time sample from the chicks during a time when the chicks appear to be receiving tetracycline. Also the chicks are introduced to the control households and chicken samples are taken 6 months after the last human samples with no description of the duration of direct contact between chickens and humans in the control group, prior to sampling the chickens. How long were the chicks exposed to the humans and the farm environment before they were sampled? What relevance does the antibiotic resistance gene screening in the poultry at 18 months have toward the study objective? This might be the place in methods section to provide more detail that informs these concerns.**

**Response:** We have added to the manuscript some of the available details requested by the reviewer, such as the duration of time the chickens were exposed to humans and the farm environment before they were sampled. Note that while chicken stool samples were obtained 18 months after randomization, this translates to chicken stool samples acquired 21 – 40 days after they were delivered to the control participants. At the time of chicken stool sampling, it was not possible to obtain concomitant human samples as that would have required transporting the participant from the villages to the clinic, which was not feasible at the time.

**Line 154 – what is meant by environmental contaminants? These samples appear to be controlling for contaminants introduced within the laboratory setting? There would be other more appropriate controls for contaminants introduced during sampling in the farm or household environments (i.e. sham samples collected on farm). Perhaps revise the wording to be more specific.
Response:** This was done as suggested. We were referring to laboratory contaminants.

**Line 173 – revise sentence, there are no “baseline” chicken samples**

**Response:** This was done as suggested. **Line 254 – again “both at baseline and follow-up” is modifying chicken samples, when there was only one time point for chicken samples**

**Response:** This was done as suggested. **Line 291 – add something to the effect of, “and people who did not care for chickens during the 12 months of human sampling (control group) harbored many of the same genes at one year.”**

**Response:** This was done as suggested. **Line 294 – revise – this wording is confusing, the humans who had direct contact with chickens at one year in the study when samples were taken was the intervention group, correct? So the control group had no direct contact with chickens at one year, yet their samples were more similar to chicken samples collected at 18 months. Was this expected? What are possible explanations for this finding?**

**Response:** We apologize for the confusion. Chicken samples were obtained from chickens distributed to the control group at the end of the study. We have revised Figure S1 so that this is clear. We have added the following. “There were greater AMR gene pattern similarities between chickens and humans who had direct interaction with the chickens in the study. The interpretation of this latter finding should be conservative, as chicken stool samples were obtained after human stool samples, and obtained only from the control group who received chickens at the end of the study. It is possible that the control group had samples more similar to their chickens either due to human to chicken transmission, or because the control group and their chickens had a common environmental source such as community wells for water.” **Line 295 – delete or revise “AMR gene patterns in both groups became more similar to AMR…” the use of the word “became” suggests a temporal association that could be perceived to imply chicken gene patterns are the driver of human gene patterns. I suggest, this sentence could also be written as chicken gene patterns at 18 months were more similar to human patterns at one year than to human patterns at base-line. Either delete or revise after analyzing the similarity of one year in both human groups to human base-line. Highlighting this comparison demonstrates the limitation of not having longitudinal chicken samples.**

**Response:** This was done as suggested. **Line 303 – revise, change to “to humans or animals” – there are examples where humans serve as a reservoir of resistant organisms that appear to be transmitted to animals, further antibiotic resistance is also detrimental to animal health**

**Response:** This was done as suggested. **Line 308 – delete or revise – “Additionally, we observed a high rate of AMR genes overall in humans and in chickens in rural Uganda” what is the comparison group – please provide a reference - higher than what other group? Urban humans and chickens in Uganda? Rural humans and chickens in other countries?**

**Response:** Our intention in stating that there is a high rate of AMR genes in both humans and chickens in Uganda is to point out the presence of numerous AMR genes, rather than to compare to existing literature (which insufficient to allow us to we can compare to other Ugandan populations and reference this). We have edited the line 308 statement in accordance with this comment.

**Line 366 – please add another limitation; the number of replicates over time (in both humans and chickens) are limited and do not represent a diversity of timing relative to chicken production cycle and antibiotic use in that cycle.**

**Response:** This was done as suggested. **Line 386 – “significant changes” – how do you define “significant” here? This conclusion can be kept if the recommended analysis comparing 1 year human to base-line human demonstrates a significant change in prevalence (frequency) – otherwise change wording to “we identified differences in the 2 time point analyzed” Of course you also identified many (more?) similarities between the 2 time points. And also there were some subjects where markers were found at baseline and not at one year. Highlight the challenges of modeling the dynamics at the level of subjects within treatment group.**

**Response:** We have revised the sentence as follows. “Here, we describe changes in the AMR gene profile in stool of humans over two timepoints”. The limitations of inferring causation in the intervention group has been highlighted elsewhere in the manuscript.

**Figure 2 – legend – source chicken crtl should be 18 months
Response:** This was done as suggested.

**In the reference section, organism genus species names should be written in italics**

**Response:** This was done as suggested.

We appreciate the reviewers comments for improving our manuscript and look forward to hearing from you about the revisions.

On behalf of all study authors,

Ana Weil and Peggy Lai
